# Correlation between clinical and pathological features of cutaneous calciphylaxis

**DOI:** 10.1371/journal.pone.0218155

**Published:** 2019-06-13

**Authors:** Puja Dutta, Kristine M. Chaudet, Rosalynn M. Nazarian, Daniela Kroshinsky, Sagar U. Nigwekar

**Affiliations:** 1 Harvard Summer Research Program in Kidney Medicine (HSRPKM), Harvard Medical School, Boston, Massachusetts, United States of America; 2 Carnegie Mellon University, Pittsburgh, Pennsylvania, United States of America; 3 Department of Pathology, Massachusetts General Hospital, Boston, Massachusetts, United States of America; 4 Department of Dermatology, Massachusetts General Hospital, Boston, Massachusetts, United States of America; 5 Division of Nephrology, Department of Medicine, Massachusetts General Hospital, Boston, Massachusetts, United States of America; Brigham and Women’s Hospital, Harvard Medical School, UNITED STATES

## Abstract

Calciphylaxis is a rare and life-threatening disease that classically manifests with painful skin lesions. It occurs mainly in patients with end-stage renal disease (ESRD) treated with dialysis, has poor outcomes, and has no FDA-approved treatment. Our cohort study aims to examine the clinical and pathological features of calciphylaxis and investigates the correlation between cutaneous clinical manifestations and histopathological findings. Data from 70 calciphylaxis patients who were evaluated at the Massachusetts General Hospital between January 2014 and April 2018 were collected from the institutional electronic database. The median age was 58 years (interquartile range [IQR]: 49–69 years), 60% were women, and 73% were of white race. Most (74%) patients reported severe pain at the time of calciphylaxis diagnosis with a median pain intensity score of 8/10 (IQR: 6–10) on a 0–10 pain scale. The median time from symptom onset to clinical diagnosis was 9 weeks (IQR: 6–16 weeks). The majority (87%) of patients presented with open necrotic wounds (advanced stage lesion) at the time of diagnosis. Common cutaneous clinical features included ulceration (79%), induration (57%), and erythema (41%), while common pathological features included cutaneous microvascular calcification (86%) and necrosis (73%). The presence of fibrin thrombi in skin biopsies was associated with pain severity (p = 0.04). The stage of a skin lesion positively correlated with the presence of necrosis on histological analyses (p = 0.02). These findings have implications for improving understanding of calciphylaxis origins and for developing novel treatments.

## Introduction

Calciphylaxis, or calcific uremic arteriolopathy, is a rare and devastating disease characterized by calcification of microvessels in the subcutaneous adipose tissue, causing painful, ischemic skin lesions [1,2]. Patients with calciphylaxis have poor clinical outcomes, with the one-year mortality rate estimated at more than 50% [3]. The exact pathogenesis of calciphylaxis is poorly understood, and there are no FDA-approved therapies for calciphylaxis [1,4].

Published literature on calciphylaxis has largely focused on risk factors and outcomes; however, data regarding cutaneous clinical features of calciphylaxis and corresponding histological features are scant. Our cohort study aims to examine the clinical and pathological features of calciphylaxis and investigates the correlation between cutaneous clinical manifestations and histopathological findings. We hypothesized that certain clinical features and previously described risk associations may predict histological features of calciphylaxis, and our study will improve the understanding of this enigmatic disease and its pathobiology, and enhance the development of novel future therapies.

## Methods

### Study patients

**Fig 1:**
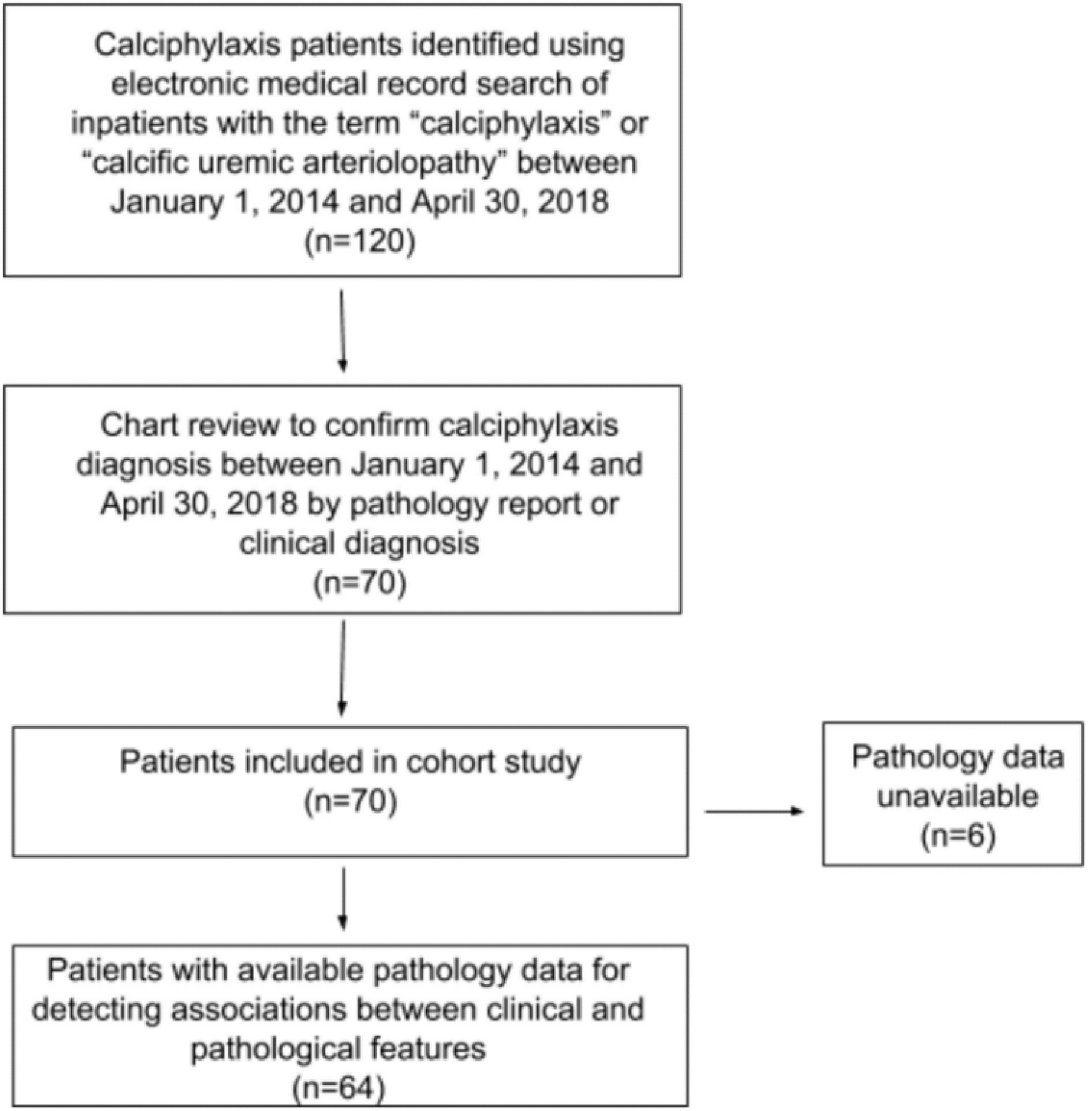
Patient selection criteria.

Data from 70 adult patients (age ≥ 18 years) with calciphylaxis who were hospitalized at the Massachusetts General Hospital between January 2014 and April 2018 and had a diagnosis of calciphylaxis via review of histopathology or clinical lesion characteristics were reviewed. Six patients did not have histopathology data available from skin biopsy, surgical debridement, or amputation and were not included in this study for detecting correlations between clinical and pathological characteristics. Fig 1 shows the flowchart of patient selection for this study.

### Study data

Data for the study patients were abstracted from the insititutional electronic database. The study protocol was approved by the Partners Institutional Review Board, and all data was anonymized before accession. These data included demographic (age, gender, and race) and clinical characteristics (body mass index (BMI), vital signs, pain score on a scale of 0–10, and cutaneous lesion characteristics), pathological findings, medications, laboratory results, and comorbid conditions. Patients having a pain score of 6 or more were classified as having “severe” pain, while patients having a pain score of 5 or less were considered as having “non-severe” pain. Cutaneous lesion characteristics included induration, ulcer, retiform purpura, livedo racemosa, black eschar, plaques, nodules, erythema, edema, lesion length, and lesion location. Histopathological characteristics included microvascular calcification (medial and intimal), necrosis, adipose tissue necrosis, perieccrine calcification, fibrin thrombi, intimal fibrosis, fibrointimal hyperplasia, ischemia, panniculitis, and location of extravascular calcium deposition in the skin (subcutaneous, deep dermal, or superficial dermis). Data for previously published risk associations for calciphylaxis (for example, warfarin, mineral bone abnormalities, diabetes mellitus, obesity, and end-stage renal disease [ESRD]), were abstracted [5–9].

Each patient’s medical record contained information for at least one lesion. We applied a previously described schema to classify calciphylaxis lesions into four stages [10]. A lesion was classified as Stage 1 if there was induration only without overlying skin changes. Stage 2 was considered to be induration with overlying skin changes (purpura) but an intact epithelium. Stage 3 was classified as having an open wound with or without blistering or obviously necrotic eschar/tissue. Stage 4 was classified as having an open wound with infection (gross purulence or cellulitis). Information for the most severe lesion reported was used for classification under a lesion stage system [10].

Each patient’s most severe skin lesion was classified using the scoring system adapted from the Mayo Clinic [4]. This was done by categorizing clinical information for a lesion as “Major” and/or “Minor” and categorizing skin biopsy information for a lesion as “Major” and/or “Minor”. Major histological criteria included medial calcification and intimal fibroplasia of pannicular arterioles with cutaneous necrosis. Minor histological criteria included extravascular calcium deposition or thrombosis of pannicular or dermal arterioles. Major clinical criteria included necrotic cutaneous ulcers over an indurated plaque, or a non-ulcerated indurated plaque in adipose-rich tissue, such as the abdominal pannus. By assessing each lesion using these criteria, we then classified each lesion as “Definite”, “Probable”, “Possible” or “None” regarding the likelihood of calciphylaxis diagnosis, using the following table adapted from the Mayo Clinic (S1 Table) [4].

### Statistical analyses

Frequency (for categorical variables), median and interquartile range (IQR) were reported (for variables not normally distributed), and mean and standard deviations (SD) were reported (for normally distributed variables). Categorical variables were compared using a Chi-squared test. Continuous variables were compared using a Wilcoxon rank sum test. To examine the associations between clinical and histological features of calciphylaxis, univariate logistic regression models were applied to compute odds ratio (OR) and 95% confidence intervals (CI). Univariate logistic regression models were also applied to examine the association between lesion stage and histological features.

All analyses were performed using the SAS statistical software (version 9.4) (SAS Institute Inc., Cary, NC). Statistical significance was set as P<0.05. To account for multiple comparisons while examining the associations between clinical and histological characteristics we applied Bonferroni correction by setting the significance cut off at <α/n (i.e. <0.001). To account for multiple comparisons while examining the associations between lesion stage and histological characteristics we applied Bonferroni correction by setting the significance cut off at <α/n (i.e. <0.01).

## Results

Table 1 provides a summary of demographic and clinical characteristics. The median age of patients at the time of diagnosis of calciphylaxis was 58 years (IQR: 49–69 years). Most patients were female (60%) and had ESRD (83%). Obesity and diabetes mellitus were present in 56% and 61% of study patients, respectively. Almost half of the patient cohort (47%) was on warfarin at the time of diagnosis of calciphylaxis. Most patients (74%) reported severe pain at the time of diagnosis, with the median pain score being 8 (IQR: 6–10) on a 0–10 pain scale. The median time from the onset of symptoms of calciphylaxis to the diagnosis was 9 weeks (IQR: 6–16 weeks). Tactile hyperesthesia was reported in 36% of patients. Neuropathic pain was reported in 27% of patients. Two of 70 study patients (3%) reported local skin trauma from injections of heparin and insulin prior to the development of calciphylaxis.

**Table 1:** Summary of clinical characteristics.

Characteristic	Cases (n = 70)
Age (yrs.)	58 (49–69)
Female Sex, %	60
White Race, %	73
Time from onset to diagnosis (weeks)	9 (6–16)
ESRD, %	83
Obesity, %	56
Diabetes mellitus, %	71
Warfarin, %	47
Pain score, (scale 0–10)	8 (6–10)
Tactile hyperesthesia, %	36
Neuropathic pain, %	27
Local skin trauma, %	3
Ulcer, %	79
Central lesion, %	73

### Cutaneous clinical manifestations

**Fig 2:**
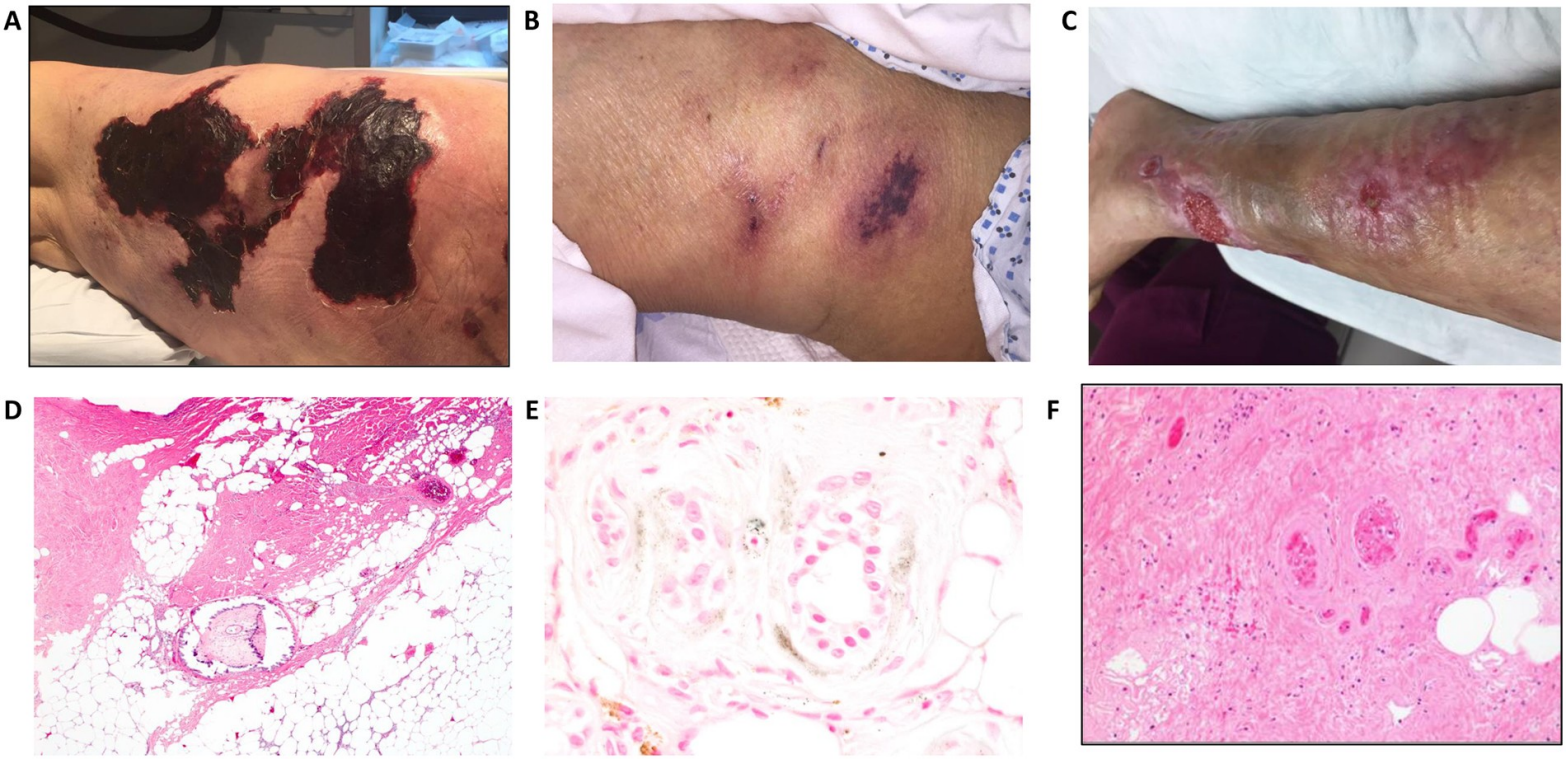
Representative images of skin lesions with various clinical and histological features. (A): Clinical image of an ulcer with black eschar and irregular violaceous border and surrounding erythema. (B): Non-ulcerated calciphylaxis lesions with plaque morphology and surrounding retiform purpura. (C): Erythematous lesions of calciphylaxis. (D): Histopathology of cutaneous calciphylaxis demonstrating ulceration, marked dermal necrosis, fibrointimal hyperplasia of a calcified medium sized vessel and subcutaneous fat necrosis with foci of acute inflammation (hematoxylin & eosin, 40x original magnification). (E): A von Kossa stain reveals presence of stippled peri-eccrine calcification (von Kossa stain, 600x original magnification). Panel F: Intravascular thrombosis in the deep dermis (hematoxylin & eosin stain, 200x original magnification).

Table 2 provides a summary of cutaneous clinical and histological features studied. Fig 2(A), 2(B) and 2(C) demonstrates representative images of skin lesions with various clinical morphologies. The presence of retiform purpura was reported in 44% of patients and erythema was seen in 41% of patients. Plaques and nodules were seen in 21% and 16% of patients, respectively. A small proportion of patients (9%) presented with livedo racemosa. Black eschar was found in 59% of patients.

**Table 2:** Association between clinical and histological features ^a^.

Histological Features (n = 64)	Clinical Features (n = 70)					
	Ulcer (79%)	Black Eschar (59%)	Plaques (21%)	Pain Severity (74%)	Erythema (41%)	Retiform Purpura (44%)
Microvascular Calcification (86%)	OR: 4.08; 95% CI: 0.80–20.73; p = 0.09	OR: 0.96; 95% CI: 0.23–3.96; p = 0.95	OR: 0.500; 95% CI: 0.11–2.32; p = 0.37	OR: 2.87; 95% CI: 0.66–12.37; p = 0.16	OR: 0.72; 95% CI: 0.19–3.20; p = 0.72	OR: 1.56; 95% CI: 0.35–6.84; p = 0.56
Necrosis (73%)	OR: 2.58; 95% CI: 0.60–11.07; p = 0.20	OR: 2.95; 95% CI:0.93–9.38; p = 0.06	OR: 0.165; 95% CI: 0.05–0.59; p = 0.006	OR: 2.02; 95% CI: 0.60–6.81; p = 0.25	OR: 2.62; 95% CI: 0.75–9.25; p = 0.13	OR: 0.28; 95% CI: 0.09–0.90; p = 0.03
Fibrin Thrombus (46%)	OR; 0.667; 95% CI: 0.16–2.75; p = 0.57	OR: 1.16; 95% CI: 0.43–3.12; p = 0.76	OR: 1.17; 95% CI:0.36–3.84; p = 0.79	OR: 3.80; 95% CI: 1.07–13.53; p = 0.04	OR: 0.83; 95% CI: 0.30–2.27; p = 0.71	OR: 1.83; 95% CI: 0.67–5.00; p = 0.23
Perieccrine Calcification (23%)	OR: 2.73; 95% CI; 0.31–23.8; p = 0.36	OR: 0.93; 95% CI: 0.29–2.97; p = 0.90	OR: 1.42; 95% CI: 0.37–5.41; p = 0.61	OR: 1.44 95%CI: 0.35–5.95 p = 0.61	OR: 1.51 95% CI: 0.47–4.85 p = 0.49	OR: 0.61; 95% CI: 0.18–2.06; p = 0.43
Subcutaneous adipose tissue necrosis (39%)	OR; 2.52; 95% CI: 0.48–13.23; p = 0.27	OR: 1.42; 95% CI: 0.52–3.94; p = 0.49	OR: 0.55; 95% CI: 0.15–2.00; p = 0.36	OR: 1.09; 95% CI: 0.34–3.50; p = 0.88	OR: 0.33; 95% CI: 0.11–1.01; p = 0.05	OR: 0.50; 95% CI: 0.17–0.41; p = 0.19

^a^. Odds ratio (OR), 95% confidence intervals (CI), and p values are reported from the examination of association between an individual clinical feature with an individual histological feature.

Based on a lesion staging system described in the methods section [5], 20% of patients had stage 2 lesions, 53% of patients had stage 3 lesions, and 27% of patients had stage 4 lesions at the time of clinical presentation. Furthermore, 79% of our cohort presented with ulcerated lesions, indicating a more severe stage of the disease. The median number of reported lesions was 1 (IQR: 1–2). The median length of a lesion was 5 cm (IQR: 1.6–9.0 cm). Most patients had centrally located lesions (73%) involving body areas such as the abdomen or thighs.

### Cutaneous histological features

The most common site of skin biopsy was the upper thigh (50%). The most common histological features were microvascular calcification (86%) and necrosis (73%). Almost half (47%) of patients had fibrin thrombi present on histological analyses. Out of the 14% of patients who did not have microvascular calcification, diagnosis was confirmed by presence of perieccrine calcification and/or fibrin thrombi, with cutaneous ulceration.

In terms of the location of calcification in the skin, the majority of microcalcification was found to be in the subcutaneous tissue (76%). Perieccrine calcification was noted in 23% of patients. Subcutaneous adipose tissue necrosis was reported in 39% of patients. Intimal fibrosis was noted in 25% of patients while fibrointimal hyperplasia was reported in 14% of patients. Fig 2(D), 2(E) and 2(F) demonstrates representative images of skin lesions with various histological features.

### Correlation between clinical and histological features

Table 2 summarizes the association between clinical and histological features. None of the associations reached the statistical significance by Bonferroni correction. Pain severity was associated with the presence of fibrin thrombi on histology (OR: 3.80, 95% CI: 1.07–13.53, p = 0.04). Fifty eight percent of patients with severe pain had skin necrosis. Although not statistically significant, microvascular calcification was more common in patients with ulcerated lesions than in those without (77% of patients with ulcerated lesion had microvascular calcification versus 9% of patients without ulcerated lesion had microvascular calcification suggesting an association between microvascular calcification and ulceration [OR: 4.08, 95% CI: 0.8–20.73, p = 0.09]). None of the associations reached the statistical significance by Bonferroni correction.

Among the five histological characteristics examined for the association, the stage of a skin lesion was associated with necrosis (OR: 4.13, 95% CI: 1.27–13.47, p = 0.02). This association, however, did not reach the statistical significance by Bonferroni correction. Additionally, the median length of a lesion was 2.5 cm (IQR: 2.0–5.0 cm) for patients reporting non-severe pain, and 5.0 cm (IQR:1.6–9.5 cm) for patients reporting severe pain.

**Fig 3:**
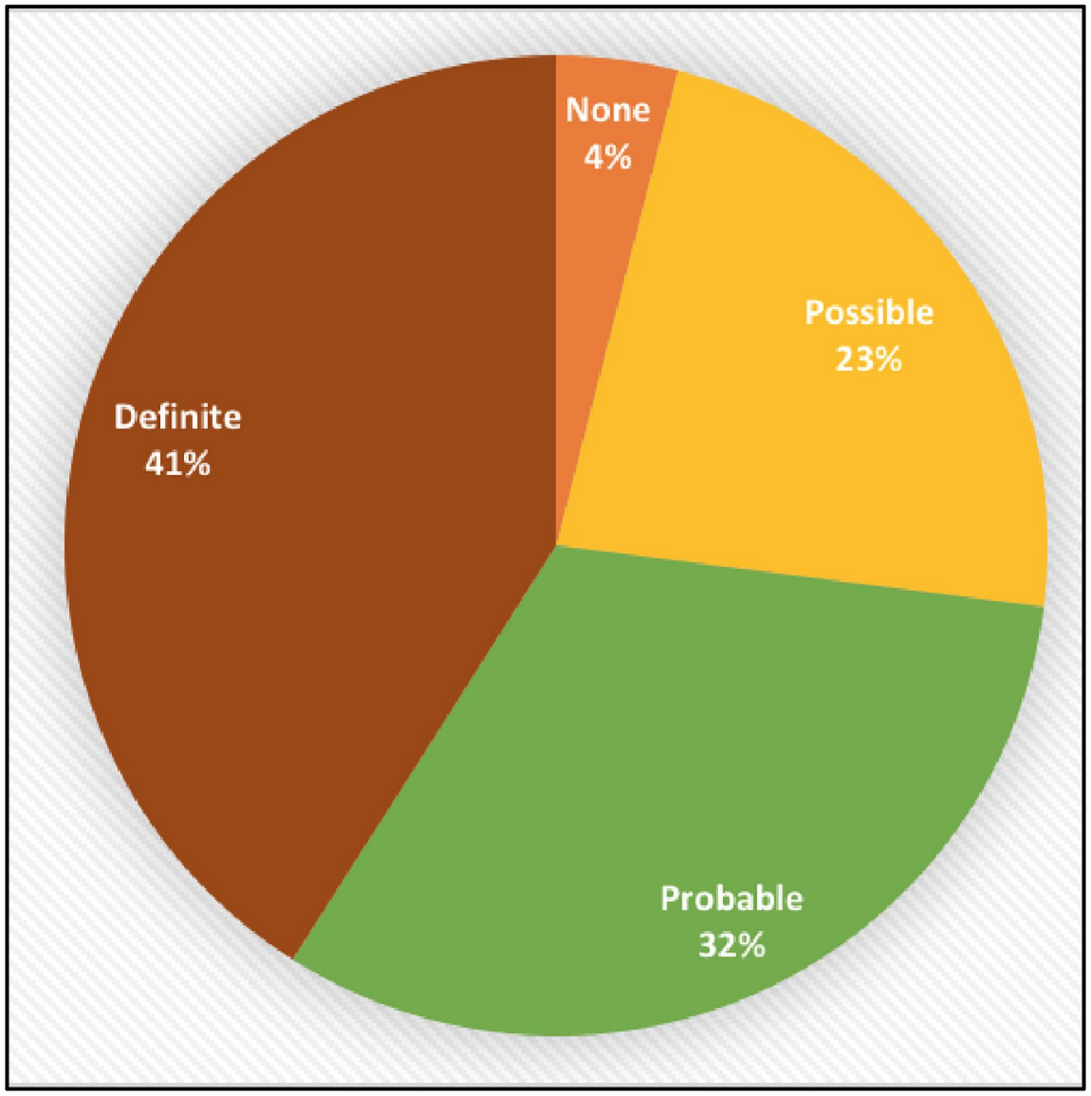
Distribution of study cohort according to the Mayo Clinic criteria [4], for likelihood of calciphylaxis diagnosis.

Fig 3 depicts the distribution of Mayo Clinic scores for our patient cohort. When a patient’s skin lesion was classified using the scoring system adapted from the Mayo Clinic [4], 32% of patient cohort were classified as having “Definite” calciphylaxis, 41% of patients were classified as having “Probable” calciphylaxis, 23% were classified as “Possible”, and 4% as “None”. In terms of pain severity and the Mayo Clinic scoring system, 56% of patients who reported severe pain were classified as having “Definite” or “Probable” calciphylaxis (n = 64, p = 0.11).

There was no significant association found between previously described risk factors for calciphylaxis (e.g. diabetes mellitus, obesity, ESRD, warfarin, etc.) and clinical or histological features of calciphylaxis. There was no association between pain severity or fibrin thrombi and mortality.

## Discussion

Our study identifies associations between cutaneous clinical features and histopathological findings in calciphylaxis patients. The presence of fibrin thrombi in skin biopsies was associated with pain severity and the stage of a skin lesion positively correlated with the presence of necrosis on histological analyses. Furthermore, microvascular calcification was found to be eight times more common in patients with ulcerated lesions than in those without.

Our analyses suggest that there is frequently a significant delay in the diagnosis of calciphylaxis. Most patients have advanced stage lesions and severe pain at the time of diagnosis. The median number of weeks between the onset of symptoms and calciphylaxis diagnosis was 9 weeks (IQR: 6–16 weeks). In a nationwide German Registry study, it was found that the median time between onset and diagnosis was 24 days (11–50 days) [11]. Furthermore, 79% of our patient cohort presented with ulcerated lesions. In the same German registry study, it was found that 63% of calciphylaxis patients had ulcerated lesions while 37% had non-ulcerated lesions [11]. By improving awareness of this disease, medical professionals may be able to achieve earlier detection and treatment initiation to improve patient outcomes.

We noted that the presence of a fibrin thrombus on histology is significantly associated with pain severity. In fact, the presence of diffuse thromboses as well as thrombosis within calcified vessels has been shown to be more common in calciphylaxis patients than in other non-calciphylaxis diagnoses [12], including nephrogenic fibrosing dermopathy, vasculitis, panniculitis, and non-specific dermal inflammation. Patients with increased propensity for thromboses formation have an increased predisposition for calciphylaxis [13,14]. Furthermore, the presence of both vascular calcification and thromboses has been shown to be six times more common in skin biopsies with high suspicion for calciphylaxis than in amputation specimens [15].

The association between fibrin thrombi and pain severity prompts us to speculate regarding an important role of fibrin in the etiopathogenesis of calciphylaxis. It is possible that cutaneous microvessels in patients with predisposition to calciphylaxis are chronically narrowed from calcification and then thrombosis triggers and dictates the onset and severity of acute pain by causing cutaneous infarction. The possible origins of fibrin and thrombus may include endothelial injury, vascular stasis, and/or local or systemic thrombophilias. Whether and how these factors disrupt the laminar blood flow in cutaneous microvessels in patients with calciphylaxis requires further mechanistic investigation.

The lack of association between the previously described risk factors for calciphylaxis and histological characteristics may suggest that there is a final common pathway leading to calciphylaxis that leads to histological characteristics independent of risk factor exposure. Furthermore, this finding may suggest that the development and progression of calciphylaxis may be distinct phases with risk factors such as warfarin triggering the disease and yet unknown factors are responsible for the disease progression and clinical and histological features.

Given that calciphylaxis patients face severe pain due to open skin lesions, finding novel therapies for pain relief is an integral part of a multi-disciplinary approach to treatment [1]. A potential future direction is developing a therapeutic to prevent fibrin thrombus formation, and thus alleviate pain. Additionally, there is data in existence showing that calciphylaxis patients with ESRD on dialysis who received anticoagulant treatment (apixaban), experienced lower mortality rates compared to established published rates for calciphylaxis patients [16]. Direct oral anticoagulants (DOACs) therapies may be a potential novel treatment option for patients with calciphylaxis and the safety and efficacy of these agents need further evaluation [17].

The Mayo Clinic scoring system implemented in this study identified 32% of our patient cohort as having “Definite” calciphylaxis. However, all 70 patients had a confirmed diagnosis of calciphylaxis by histopathology or clinical suspicion suggesting that additional evaluation of its sensitivity and specificity and validation in prospective studies is required.

There was no significant association between warfarin use and the pathological variables studied. Warfarin is a risk factor for developing calciphylaxis [1,2,12,16,18,19]. It plays a role as a vitamin K antagonist, and vitamin K is an inhibitor of calcification [1,2,16]. In this way, since warfarin acts as a vitamin K antagonist, it impedes the inhibition of calcification, and thus leads to vascular calcification. However, given that there is no association between warfarin use and all pathological variables studied, this suggests that regardless of warfarin usage, there is a final common pathway of pathogenesis of this disease.

Our study has limitations. A larger sample size may strengthen the significance of some of the notable findings between clinical characteristics and histopathological findings. In that regard, it is important to note that none of the associations were statistically significant after applying the correction for multiple comparisons. Correlation between these variables does not necessarily imply causation. Our study was a retrospective study, and we restricted our study to patients hospitalized at our institute, and thus future studies that include outpatients with calciphylaxis are needed. Investigation of clinical and histological correlation will not only improve our understanding of this rare yet life-threatening disease, but also help design experimental studies to uncover pathobiology, and our ability to develop effective therapeutic treatments. Future larger studies should examine whether and how calciphylaxis risk factors influence the clinical presentation and histological features.

In conclusion, the majority of our patient cohort were middle-aged, had ESRD, and presented to the hospital with open, ulcerated lesions with severe pain, indicating a potential delay in diagnosis. Clinical characteristics may correlate with histopathological findings in calciphylaxis patients. The presence of fibrin thrombi is associated with pain severity, which opens a potential avenue for a future therapeutic target. These preliminary findings need confirmation in a larger prospective study.

## Supporting information

S1 Table Mayo Clinic criteria for diagnosis of calciphylaxis [4]. (DOCX)
