# Supplementary material for: Correlation between clinical and pathological features of cutaneous calciphylaxis
Source: PLoS One. 2019 Jun 13;14(6):e0218155. doi: 10.1371/journal.pone.0218155 (PMC6564670; doi:10.1371/journal.pone.0218155)
Supplement: S1 Table — (DOCX) [file pone.0218155.s001.docx]

**Table S1. Mayo Clinic criteria for diagnosis of calciphylaxis [4]**

|  | Clinical Criteria | | | | | |
| --- | --- | --- | --- | --- | --- | --- |
| Skin Biopsy Criteria | 2 Major | 1 Major | 3 Minor | 2 Minor | 1 Minor | None |
| 1 Major | Definite | Definite | Definite | Definite | Definite | Probable |
| 2 Minor | Definite | Definite | Definite | Probable | Possible | Possible |
| 1 Minor | Definite | Probable | Probable | Possible | Possible | No |
| ND or none | Definite | Probable | Probable | Possible | No | No |
